# Expression of Programmed Death Receptor 1 (PD-1) Gene and Its Ligand (PD-L1) in Patients with Laryngeal Cancer

**DOI:** 10.3390/biom11070970

**Published:** 2021-07-01

**Authors:** Andrzej Kowalski, Katarzyna Malinowska, Jurek Olszewski, Hanna Zielińska-Bliźniewska

**Affiliations:** 1 Department of Otolaryngology, Laryngological Oncology, Audiology and Phoniatrics, Medical University of Lodz, 90-549 Lodz, Poland; jurek.olszewski@umed.lodz.pl; 2 Department of Allergology and Respiratory Rehabilitation, Medical University of Lodz, 90-725 Lodz, Poland; katarzyna.malinowska@umed.lodz.pl (K.M.); hanna.zielinska-blizniewska@umed.lodz.pl (H.Z.-B.)

**Keywords:** programmed death receptor 1 (PD-1), programmed death-ligand 1 (PD-L1), laryngeal cancer

## Abstract

(1) Background: The interaction of the programmed death receptor (PD-1) with its ligand 1 (PD-L1) allows cancer cells to escape from the control of the immune system. Research evaluating the expression of immune checkpoint genes in the tissues of laryngeal tumors may contribute to the introduction of new effective immunotherapeutic methods in this group of neoplasms. The aim of this study was to evaluate the expression of the gene for the programmed death receptor (PD-1) and its ligand (PD-L1) in laryngeal tumors (T1, T2, T3) in patients without lymph node involvement and distant metastases. (2) Methods: The study included 73 patients: 39 of them were diagnosed with carcinoma planoepiteliale keratodes (study group) and 34 with nasal septal deviation undergoing septoplasty (control group). Biological material for molecular tests (Real time PCR) was collected during surgical procedures. Furthermore, all study participants completed a questionnaire regarding, among others, smoking and body weight. (3) Results: Gene expression for programmed death receptor 1 (PD-1) and its ligand 1 (PD-L1) was, statistically, significantly higher (*p* < 0.0001) in tumor tissue than in unchanged mucosa. Moreover, it was found that the greater the tumor size, the higher the expression level of the tested molecules. (4) Conclusions: Although further research on the role of the PD-1/PD-L1 pathway in laryngeal tumors is necessary, the presented reports are promising and may constitute a contribution to considerations on the introduction of targeted immunotherapy with anti-PD1 and anti-PD-L1 monoclonal antibodies in the treatment of these tumors.

## 1. Introduction

The immune system plays an extremely important role in the body defense not only against external pathogens but also against its own cells, which display certain abnormalities. Such abnormal cells include, among others, senescent, virus-infected, and neoplastic cells. Immunological mechanisms aim to eliminate defective cells as quickly as possible with simultaneous preservation of tolerance to antigens on the surface of its own cells (autoantigens). Programmed death receptor 1 (PD-1; CD279) is responsible for maintaining this balance.

Programmed death receptor 1 (PD-1), encoded by the PDCD-1 gene, located on the long arm of chromosome 2 (locus 2q37.3) [1], is classified as an oncogene [2]. Increased expression of PD-1, associated with the activation of T lymphocytes, B lymphocytes and monocytes, is also characterized by cell exhaustion, which is associated with the loss of cytotoxic T lymphocyte function. PD-1 regulates the immune response by inhibiting the activation of lymphocytes [3,4]. PD-1 belongs to the CD28 family. It has two ligands. The first ligand, PD-L1, inhibits autoimmune responses by negative regulation of proliferation and production of cytokines by T cells [5,6]. In 2001, a second ligand for this receptor (PD-L2) was discovered, also having an inhibitory effect on T lymphocytes [4,6].

The interaction of the PD-1 receptor with its ligands is an example of the mechanism of escape of tumor cells, from the control of the immune system, by intensification of the negative signal transmitted to tumor-infiltrating lymphocytes, which impairs their function [6,7,8]. The expression of negative regulators of the immune system (including the PD-1/PD-L1 pathway) allows for the creation of an immunosuppressive microenvironment [9].

In recent years, there has been a significant development of immunotherapeutic methods in the treatment of neoplastic diseases. The most commonly used are monoclonal antibodies, among others, blocking PD-1 molecules, present on the surface of T lymphocytes and PD-L1 molecules, found on the surface of tumor cells, and on the surface of immune cells, infiltrating the tumor microenvironment. Therapy with anti-PD-1 and anti-PD-L1 antibodies leads to the induction of immune response and to the destruction of tumor cells [9,10]. It is used in the treatment of many cancers, including melanoma, thyroid cancer, lung cancer, and breast cancer [9]. So far, targeted immunotherapy has not been used in the treatment of laryngeal cancer. However, research aimed at assessing the expression of immune checkpoint genes in the tissues of laryngeal tumors may contribute to the introduction of new effective methods of treatment.

The aim of our study was to evaluate the expression of the gene for the programmed death receptor (PD-1) and its ligand (PD-L1) in laryngeal tumors (T1, T2, T3) in patients without lymph node involvement and distant metastases.

## 2. Materials and Methods

### 2.1. Study Participants/Characteristics of the Investigated Group

A total of 73 patients (12 F, 61 M) aged 32–91 years (mean age 58.55 ± 13.80 years) participated in the study. The insignificant majority (50.68%) were subjects with normal body weight (BMI < 25), who smoked (58.90%) on average, for over 33 years, about 20 cigarettes a day (Table 1).

The study group included 39 patients diagnosed with laryngeal cancer (4 F, 35 M), mean age 67.97 ± 8.93 years, most of them (56.41%) with normal body weight. In this group, the vast majority of participants (89.74%) were smokers, who had smoked about 20 cigarettes a day for over 35 years. In the majority of patients, the tumor stage was assessed as T1N0M0 (64.10%) (Table 1).

The control group consisted of 34 healthy subjects (8 F, 26 M), mean age 47.73 ± 9.92 years, without inflammatory changes in the paranasal sinuses, with a deviation of the nasal septum in whom, during septoplasty procedure, a fragment of the nasal septum mucosa was collected for molecular testing. In this group, 55.88% of patients had abnormal body weight (overweight or obesity), and only 23.53% of them confirmed smoking (Table 1).

Both groups (study group and control group) did not differ statistically significantly in terms of sex (*p* = 0.1130) or body weight assessed by the body mass index (BMI) (*p* = 0.3200). Men dominated in both groups (89.74% and 76.47%, respectively). Patients with laryngeal tumor were older (*p* < 0.0001; 67.97 ± 13.80 years vs. 47.73 ± 8.93 years) and smoked more frequently (*p* < 0.0001; 89.74% vs. 23.53%) (Table 1). However, further analysis showed no correlation between age and the expression of the studied genes.

Written informed consent was obtained from each subject according to the study protocol that had been approved by the Bioethical Committee of the Medical University of Lodz No: RNN/140/15/KE + KE/433/21

### 2.2. Survey Data

Survey data, i.e., age, gender, weight, height, and information regarding smoking, were obtained from all study participants. Patients answered questions about whether they smoked cigarettes. If the answer was yes, they also had to state for how long and how many cigarettes daily. The age and height of the respondents were used to calculate the body mass index (BMI) according to the formula:

BMI = weight [kg]/(height [cm])^2^ (1)

Body weight was assessed on the basis of the BMI criteria according to the World Health Organization (WHO), which indicates that the correct body weight is a BMI in the range of 18.5–24.99. Body mass index > 25 indicates overweight and >30-obesity [11].

### 2.3. Assessment of Tumor Advancement and Collection of Samples

Laryngeal tumors were assessed by videolaryngostroboscopy (VLS) and by histopathological examination (taking a sample of the lesion in direct microlaryngoscopy). The obtained test result in all patients was: planoepithelial carcinoma keratodes (carcinoma planoepiteliale keratodes).

Neck USG, CT with contrast, and chest X-ray were performed in patients to assess lymph nodes. No patients in the study group had enlarged lymph nodes nor lung metastases.

According to the TNM classification of laryngeal tumors (according to AJCC, 2017), 25 patients (64.10%) had T1a N0 M0, a tumor limited to one vocal fold with fold movement, without lymph node metastases and distant metastases. In 7 (17.95%) patients, T2 N0M0 was found, i.e., the tumor involved the entire vocal fold, reached the anterior commissure, with non-movement of the vocal fold and in the other 7 (17.95%) patients, T3 N0M0 was found,—tumor confined to the larynx with non-movement of vocal folds [12].

Samples of the tumor tissue were collected during surgical procedures of cordectomy (in the T1 and T2 stages of advancement) and total laryngectomy (in T3 stage). Each sample was evaluated by the pathologist, who diagnosed carcinoma planoepitheliale laryngis- laryngeal squamous cell carcinoma (LSCC). Only a part of a sample was used for further molecular tests.

### 2.4. Molecular Analysis/Assessment of PD-1 and PD-L1 Gene Expression

Immediately after collection, the tumor fragment was placed in Eppendorf tubes with 1 mL of RNAlater fluid (Qiagen, Hilden, Germany), preventing RNA degradation, and frozen at −20 °C. Then, it was sent to the Central Laboratory of the Medical University of Lodz to evaluate the expression of PD-1 and PD-L1 genes.

#### 2.4.1. Total RNA Extraction

Tissues were removed into 200 µL of RNAlater solution (Ambion, Austin, TX, USA) and stored at −20 °C until RNA isolation. Total RNA extractions were carried out using RNeasy Mini kit (Qiagen, Hilden, Germany) and an TissueRuptor homogenizer (Qiagen, Hilden, Germany), according to the manufacturer’s instructions. RNA content and purity were measured using PicoDrop spectrophotometer (Picodrop Limited, Radnor, PA, USA). The quality of RNA was analyzed by measuring the ratio of absorptions at 260/280 nm and samples with A260/A280 coefficient above 1.8 were selected for further analysis. The purified total RNA was immediately used for cDNA synthesis or stored at −800 °C.

#### 2.4.2. Reverse Transcription

Generation of cDNA was performed with Maxima First Strand cDNA Synthesis Kit (Thermo Fisher Scientific Inc., Foster City, CA, USA) according to the protocol of the manufacturer, and 1 μg of total RNA was used as starting material. Reverse transcription was performed in conditions optimized for this kit (25 °C for 10 min, 50 °C for 30 min, 85 °C for 5 min). The cDNA samples were kept frozen at −20 °C.

#### 2.4.3. Real Time PCR

TaqMan gene expression experiments were performed in 10 μL reactions, including 50 ng cDNA, 5 μL TaqMan Universal PCR Master Mix, and 0.5 μL appropriate TaqMan Gene Expression Assay (20×). Specific Pre-made TaqMan assays used in this study were: programmed cell death 1 (PDCD1, Applied Biosystems, Foster City, CA, USA, code-Hs01550088_m1), programmed cell death 1ligand 1 (PDCD1LG1, Hs01125301_m1), and beta actin (ACTB, Hs01060665_g1) as the endogenous control. TaqMan PCR assays were performed on a 7900HT Fast Real-Time PCR System (Applied Biosystems, Foster City, CA, USA) in 96-well PCR plates (Nippon Genetics Europe GmbH, Düren, Germany). The reactions were incubated in a 96-well plate at 950 °C for 20 s, followed by 40 cycles each for 3 s at 95 °C and 30 s at 60 °C. Expression values were calculated using SDS 2.3 Software (Applied Biosystems, Foster City, CA, USA) and RQ values were calculated according to the ΔΔCt method.

### 2.5. Statistical Analysis

Categorical variables were described using absolute numbers and percentages. Numerical traits were depicted by their mean and standard deviation values. The normality of distribution was appraised by using the Shapiro–Wilk test. The homogeneity of variances was tested by using Levene’s test. One way and multifactor ANOVA (for normally distributed variables) or generalized linear models (for non-normally distributed ones) were used to assess differences in numerical traits. The Pearson product-moment correlation coefficient was computed for two numerical variables bivariate relationships. A level of *p* < 0.05 was deemed statistically significant. All the statistical procedures were performed by using Stata/Standard Edition, release 14.2 (StataCorp LLC, College Station, TX, USA).

## 3. Results

**Figure 1:**
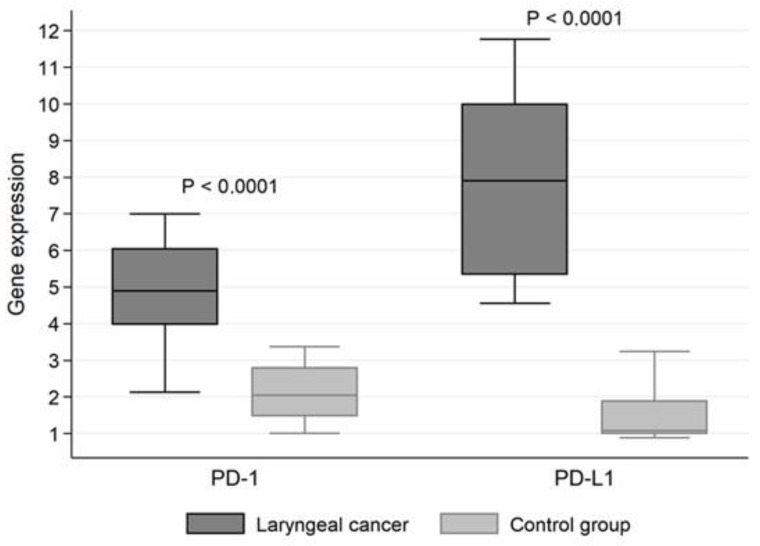
Comparison of programmed death receptor 1 (PD-1) and its ligand 1 (PD-L1) gene expression.

Programmed death receptor 1 (PD-1) gene expression was statistically significantly higher (*p* < 0.0001) in tumor tissue than in unchanged mucosa (mean PD-1 mRNA expression 5.062 ± 1.382 vs. 2.047 ± 0.705). Similarly, for programmed death receptor 1 (PD-L1) ligand, mRNA expression was significantly higher (*p* < 0.0001) in tumor tissue (8.117 ± 2.593) than in the control group (1.389 ± 0.663) (Figure 1).

**Figure 2:**
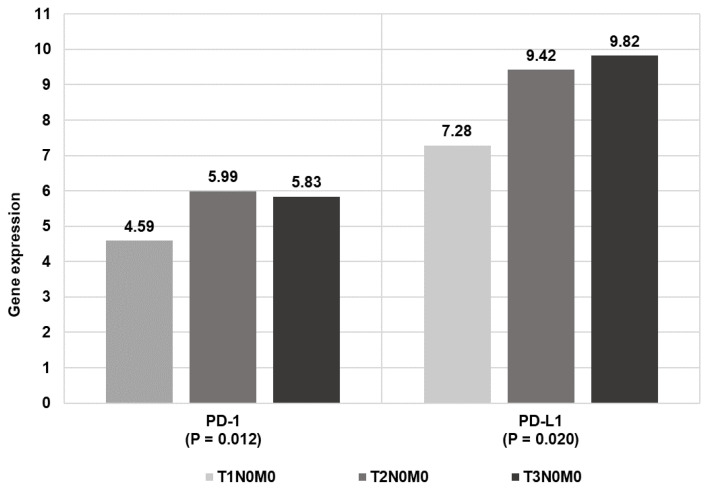
PD-1 and PD-L1 mean expression in patients with laryngeal cancer by TNM classification.

The higher the tumor staging, the higher the expression level of both the programmed death receptor (PD-1) gene and its ligand 1 (PD-L1) (Table 2 and Figure 2).

There was found a statistically significant positive correlation between PD-1 and PD-L1 expression level in the study group. The correlation coefficient, *r* = 0.650 (95% CI: 0.421, 0.801), denotes the strong positive relationship between the two analyzed expression levels.

PD-L1 expression was statistically significantly greater in patients who smoked cigarettes, *p* = 0.0095 overall and *p* = 0.0231 in the control group. In the study group, taking into account that 35 out of 39 did smoke, the dependence was neither statistically meaningful nor mentionable. The number of cigarettes the subjects smoked per day and their years of smoking were not statistically significant predictors of PD-1 and PD-L1 expression in both study groups.

Our study demonstrated that BMI had no influence on the expression of the studied genes. Correlation coefficients considering BMI and gene expression levels, in the studied patients altogether: PD-1, *r* = −0.080 (*p* = 0.5006), PD-L1, *r* = −0.141 (*p* = 0.2331). The correlation coefficients of BMI and gene expression in the group of subjects with laryngeal cancer are as follows: PD-1, *r* = 0.030 (*p* = 0.8582), PD-L1, *r* = −0.051 (*p* = 0.7564), and in the control group: PD-1, *r* = −0.264 (*p* = 0.1318), PFD-L1, *r* = −0.141 (*p* = 0.2331).

Similarly, the age of the patients had no effect on gene expression for the tested immune checkpoint genes (*p* = 0.442 for PD-1 and *p* = 0.115 for PD-L1).

## 4. Discussion

Many studies have revealed an increased expression of PD-1 receptor and its ligands in neoplastic cells, including, among others, in lung, prostate, colon, esophageal, stomach, kidney, breast, ovarian, bladder, head, and neck cancers [13,14,15]. Wang et al. [16] conducted a study in 70 patients, including 60 with laryngeal squamous cell carcinoma (LSCC) and 10 with hypopharyngeal squamous cell carcinoma (HSCC). They found that PD-1 and PD-L1 expression in LSCC and HSCC was significantly higher than in healthy tissues. They also showed a relationship between the location of the tumor and the level of PD-L1 expression. PD-L1 expression in LSCC was significantly higher than in HSCC (*p* < 0.05), moreover, PD-L1 expression in tissues of LSCC within glottis or subglottis was significantly higher than in the tumors of supraglottis (*p* < 0.05). This may be due to the rich lymphatic vasculature of the supglottis and may be associated with inflammatory factors such as INF-gamma [16]. Our study also confirmed the increased expression of PD-1 and PD-L1 genes in tissues of laryngeal tumors.

Research emphasizes the relationship between the expression of PD-1/PD-L1 pathway molecules in tumor tissues and the prognosis. It has been indicated that high PD-L1 expression on kidney cancer cells or infiltrating T lymphocytes is associated with an aggressive course of the disease and an over 4-fold higher risk of death [17]. A similar relationship of survival with tumor PD-L1 expression was observed in ovarian cancer, where additionally increased ligand 1 (PD-L1) expression negatively correlated with the amount of CD8+ T cells in the ovary [18]. In tumors with increased expression of PD-L1, apart from the inhibition of T lymphocytes activity, a greater resistance of tumor cells to apoptosis was also found, which indicates an additional defense mechanism of tumors using the PD-1/PD-L pathway [5,18,19].

Recent studies emphasize that the level of PD-L1 in tumor cells or tumor microenvironment cells is a promising biomarker for the prognosis of cancer patients [16,20]. The tumor staging system is also important for them. Currently, the TNM system is still the most frequently used classification for staging and prognosis in cancer.

Many authors indicate that high PD-1 and PD-L1 expression may promote tumor progression. The research have shown the positive correlation between the expression of these molecules and the presence of lymph node involvment, metastases, and tumor stage [16,20]. Our study confirmed that the higher the tumor advancement, the higher the gene expression for immune checkpoint genes. However, it should be remembered that, without the use of immunotherapy, it may result in a worse prognosis. The situation of using targeted monoclonal antibodies may result in a better therapeutic response to this type of treatment. On the other hand, Birtalana et al. [21] concluded that PD-L1 expression on immune cells indicates better prognosis in laryngeal squamous cell carcinoma and in HPV-negative head and neck squamous cell carcinoma.

Studies on the survival of cancer patients with high PD-L1 levels are not conclusive. Not all of them indicate a reduced survival rate in this group of patients. These conflicting observations may be due to the fact that researchers often do not distinguish the level of PD-L1 in neoplastic cells from the level of PD-L1 in the tumor microenvironment [19,22]. The expression of PD-1 and PD-L1 on tumor cells, freshly collected in vivo, was found to be much higher than on cells cultured in vitro, which, due to the influence of cytokines, may suggest that it is the tumor microenvironment that has a large impact on shaping the expression of elements of the PD-1/PD-L1 pathway [9,22,23,24].

The Food and Drug Administration (FDA) has approved two immunotherapeutic drugs targeting PD-1 -pembrolizumab and nivolumab for the treatment of relapsed and metastatic HNSCC (Head and Neck Squamous Cell Carcinoma. In 2019, pembrolizumab was approved for the treatment of inoperable relapses/metastases in patients with HNSCC [25,26]. Further studies are needed to assess whether such immunotherapy would be beneficial and safe in less advanced tumors to, for example, prevent relapses.

## 5. Conclusions

Gene expression for the programmed death receptor (PD-1) and its ligand 1 (PD-L1) is significantly increased in laryngeal tumor tissues. The higher the expression, the higher the local tumor stage advancement. Although further research on the role of the PD-1/PD-L1 pathway in laryngeal tumors is necessary, the presented reports are promising and may contribute to considerations on the introduction of targeted immunotherapy, with anti-PD1 and anti-PD-L1 monoclonal antibodies, in the regular treatment of this group of tumors. It may be suspected that patients with higher expression of PD-1/PD-L1 pathway elements on the surface of tumor cells, and in its microenvironment, will respond better to targeted immunotherapy. These molecules might serve as prognostic biomarkers. It is also worth noting that the expression of immune checkpoint genes, which helps certain cancers escape from the control of the immune system, may also depend on certain external factors, such as smoking. Hence, further studies on the role of the immune system in laryngeal cancer are so important.

## Figures and Tables

**Table 1:** Baseline characteristics of the study cohort and study groups.

Analyzed Trait	Laryngeal Cancer (*n* = 39)	Control Group (*n* = 34)	Overall (*n* = 73)	*p*-Value **
	*n*/M (%/SD)	*n*/M (%/SD)	*n*/M (%/SD) *	
Gender:				
- Female	4 (10.26)	8 (23.53)	12 (16.44)	=0.1130
- Male	35 (89.74)	26 (76.47)	61 (83.56)	
Age (years)	67.97 (8.93)	47.73 (9.92)	58.55 (13.80)	<0.0001
BMI (kg × m^−2^)	25.24 (3.13)	26.03 (3.58)	25.61 (3.35)	=0.3200
Body weight categories:				
- normal weight	22 (56.41)	15 (44.12)	37 (50.68)	=0.2947
- overweight/obesity	17 (43.59)	19 (55.88)	36 (49.32)	
TNM grading:				
- T1N0M0	25 (64.10)			
- T2N0M0	7 (17.95)	N/A	N/A	N/A
- T3N0M0	7 (17.95)			
Smoking status (“Yes”)	35 (89.74)	8 (23.53)	43 (58.90)	<0.0001
Duration of smoking (years)	35.20 (6.84)	26.25 (9.16)	33.53 (8.02)	=0.0205
Number of pieces smoked per day	19.71 (1.18)	20.00 (0.00)	19,77 (1.06)	=0.4938

* Abbreviations: n—number; M—mean; %—percentage; SD—standard deviation; N/A-not applicable; ** Univariate analyses.

**Table 2:** PD-1 and PD-L1 expression in patients with laryngeal cancer by TNM classification.

Gene Expression	T1N0M0	T2N0M0	T3N0M0
	M (SD)	M (SD)	M (SD)
PD-1	4.59 (1.30)	5.99 (1.08)	5.83 (1.24)
PD-L1	7.28 (2.31)	9.42 (2.74)	9.82 (2.33)

## Data Availability

The data analyzed in the study are available upon request to the authors of the article.
